# Detection of Low Levels of DNAJB1::PRKACA Fusion KinaseRequires the Utilization of Sensitive Assays and CarefulMethodological Planning. Reply to Palaz et al. Independent Multi-Cohort RNA-Seq Analysis Does Not Support Recurrent DNAJB1::PRKACA Fusion in Hepatoblastoma or Biliary Atresia. Comment on “Fleifil et al. DNAJB1-PKAc Kinase Is Expressed in Young Patients with Pediatric Liver Cancers and Enhances Carcinogenic Pathways. *Cancers* 2025, *17*, 83”

**DOI:** 10.3390/cancers18162706

**Published:** 2026-08-21

**Authors:** Yasmeen Fleifil, Ruhi Gulati, Katherine Jennings, Alexander Miethke, Alexander Bondoc, Gregory Tiao, Rebekah Karns, Lubov Timchenko, Nikolai Timchenko

**Affiliations:** 1 Division of General and Thoracic Surgery, Cincinnati Children’s Hospital Medical Center, Cincinnati, OH 45229, USAalex.bondoc@cchmc.org (A.B.); 2 Department of Neurology, Cincinnati Children’s Hospital Medical Center, Cincinnati, OH 45229, USA; 3 Department of Gastroenterology, Hepatology & Nutrition, Cincinnati Children Hospital Medical Center, Cincinnati, OH 45229, USA; 4 Department of Pediatrics, University of Cincinnati College of Medicine, Cincinnati, OH 45229, USA; 5 Department of Surgery, University of Cincinnati College of Medicine, Cincinnati, OH 45229, USA; 6 Division of Abdominal Transplantation, Stanford University, Palo Alto, CA 94304, USA

**Keywords:** fibrolamellar HCC, hepatoblastoma, biliary atresia, DNAJB1::PRKACA

## Abstract

In 2024, we published a paper which described the identification of the fusion DNAJB1::PRKACA kinase in young patients with aggressive hepatoblastoma (HBL) and with biliary atresia (BA). In our study, we used sensitive molecular and cellular techniques and found that about 70% of our analyzed HBL samples showed varying levels of DNAJB1::PRKACA expression. In total, 15–20% of those fusion-positive HBL samples had DNAJB1::PRKACA levels comparable to those observed in FLC, whereas the remaining samples exhibited lower kinase levels. Palaz et al. analyzed five HBL datasets and one BA RNA-Seq dataset with Arriba and found no DNAJB1::PRKACA fusion transcript. Based on their analysis, the authors concluded that DNAJB1::PRKACA is not expressed in HBL patients. They further stated that the identification of DNAJB1::PRKACA fusion in either HBL or BA requires orthogonal molecular validation to confirm the fusion event at the DNA or RNA level. Therefore, we conducted Sanger sequencing on RT-PCR products from several fusion-positive HBL samples and three BA samples. Both HBL and BA cases showed the presence of the DNAJB1::PRKACA fusion transcript, identical to that detected in FLC. These results verify that DNAJB1::PRKACA is present in some HBL and BA patients, as has already been demonstrated using sensitive molecular and cellular techniques. Arriba analysis of current RNA-Seq data may not be sensitive enough for detecting low DNAJB1::PRKACA levels in HBL cases, especially if they are mixed with fusion-negative HBL specimens. Thus, we found that several independent methods, such as immunoanalysis and RT-PCR sequencing, effectively detect DNAJB1::PRKACA in HBL and BA cases. Our study also highlights that detecting low levels of DNAJB1::PRKACA requires individual analysis of HBL and BA patients within the same study, using FLC as the control.

## Simple Summary

Our prior study found that the fusion DNAJB1::PRKACA kinase, originally found in fibrolamellar hepatocellular carcinoma (FLC) and seen in Oncocytic Pancreatobiliary Neoplasms (IOPNs), is also present in some young patients with hepatoblastoma (HBL) and biliary atresia (BA). Palaz et al. analyzed several HBL and BA RNA-Seq datasets with Arriba but did not detect the DNAJB1::PRKACA fusion transcript, likely because the assay lacks sufficient sensitivity for identifying fusion events in heterogeneous HBL and BA cases. We present evidence that, using Sanger sequencing on RT-PCR products, the DNAJB1::PRKACA fusion transcript was detected in HBL and BA patients, identical to those found in FLC. Our findings indicate that detecting DNAJB1::PRKACA transcripts in HBL patients, even at low fusion-kinase levels, requires careful evaluation of each patient’s specimen. This process involves several sensitive tests within the same study, focusing on fusion detection, and using FLC controls for accuracy.

*Fibrolamellar Hepatocellular Carcinoma (FLC): Fusion Kinase DNAJB1::PRKACA Is Highly Expressed in FLC Patients but Is Also Detected in Other Cancers*

FLC is a liver cancer found in adolescents and young adults, with no effective treatments currently available other than surgical resection [1]. In 2014, Simon’s group identified that FLC is linked to the deletion of a 400 kb DNA segment, resulting in the formation of the DNAJB1::PRKACA fusion protein [2]. Further studies on FLC have shown that DNAJB1::PRKACA is a contributor to the development of FLC [3,4], and analysis of mouse models expressing DNAJB1::PRKACA confirmed the critical role of the fusion kinase in FLC [5,6]. Despite this, some studies have reported FLC development without the DNAJB1::PRKACA fusion kinase [7]. A recent report found that overexpressing native PRKACA yields an FLC-like transcriptome signature without requiring the DNAJB1::PRKACA fusion, emphasizing the complexity of the molecular mechanisms occurring in FLC [8]. Notably, although DNAJB1::PRKACA is highly expressed in FLC patients, its expression is not specific to FLC and has been reported in several non-FLC cancers. At least nine reports have described DNAJB1::PRKACA expression in non-FLC cancers [9]. Several studies have detected DNAJB1::PRKACA in intraductal oncocytic pancreatic neoplasms (IOPNs), supporting this fusion as a molecular hallmark of IOPN [10]. Therefore, researchers should use caution when defining DNAJB1::PRKACA as a marker of FLC.

*Sanger Sequencing of RT-PCR Products Confirmed the Expression of DNAJB1::PRKACA in HBL Patients and in Patients with Biliary Atresia*

Our *Cancers* paper reported the presence of DNAJB1::PRKACA in some young HBL and BA patients [9]. To further investigate the presence of DNAJB1::PRKACA in HBL and BA samples, we conducted Sanger sequencing of RT-PCR products. The samples analyzed were three of the previously described fusion-positive HBL cases (97, 111 and 117) and three biliary atresia samples, which were previously described in our *Cancers* paper [9], and were compared to positive FLC controls.

**Figure 1:**
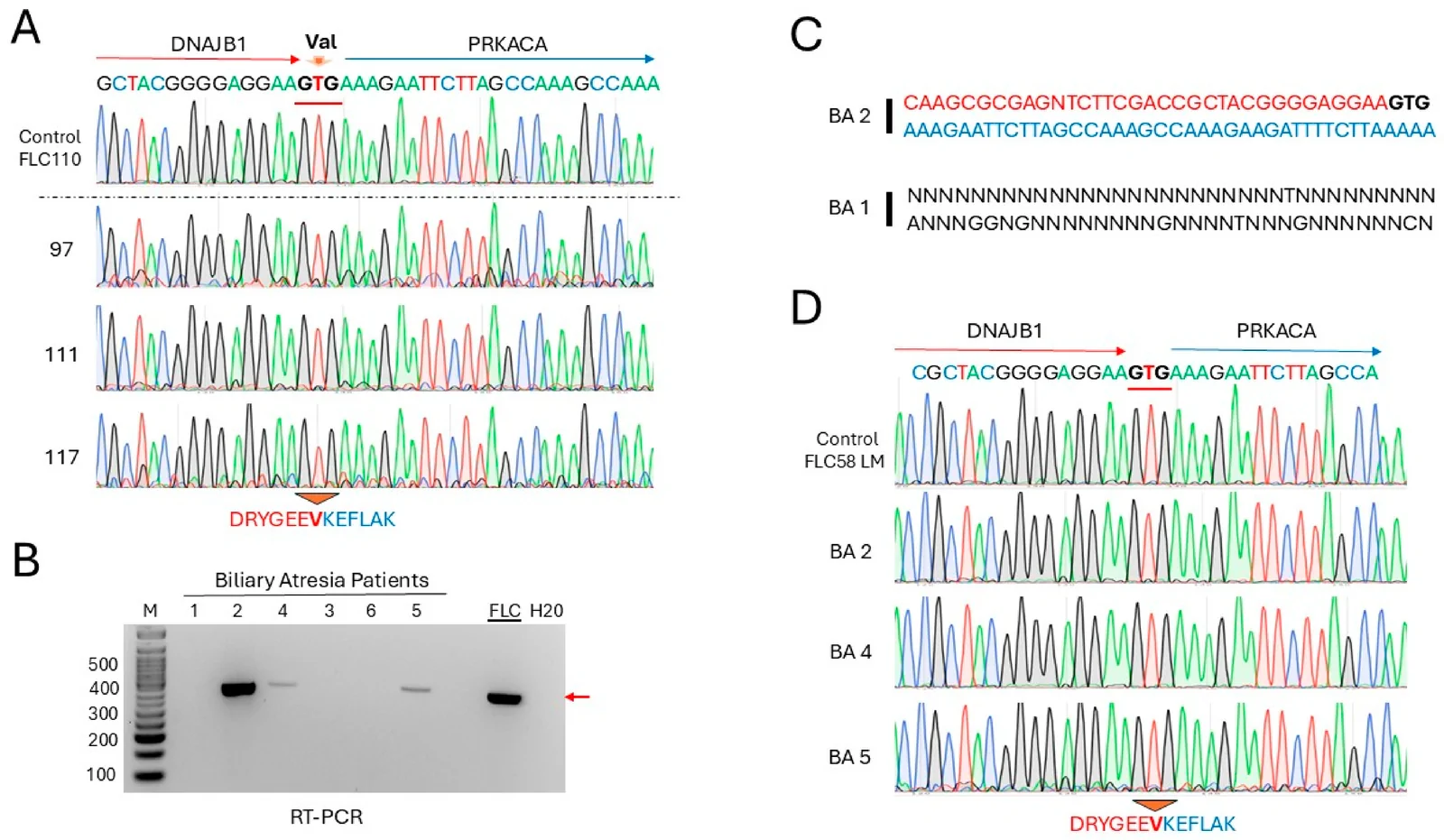
Sanger sequencing results. (**A**) Nucleotide peak alignment of sequencing for the FLC control and HBL (97, 111, 117) described in our *Cancers* paper [9]. The upper read shows the nucleotide sequence in the fusion region. The underlined GTG codon connects DNAJB1 to PRKACA. The bottom image shows the amino acid sequence of the junction region. (**B**) Detection of fusion transcripts by RT-PCR in patients with biliary atresia (BA). Patients 2, 4, and 5 express the fusion kinase, whereas patients 1, 3, and 6 are negative. FLC is a positive control. The no-template control (H2O) confirms the absence of contamination. (**C**) Sanger sequencing of RT-PCR products from fusion-positive BA 2 and fusion-negative BA 1 confirms the specificity of fusion sequence detection. Red indicates the DNAJB1 sequence and blue indicates the PRKACA sequence. (**D**) Nucleotide peak alignment of sequencing results for BA patients 2, 4, and 5. The RT-PCR product from FLC58 lung metastasis is shown as a positive control. The amino acid sequence of the junction region is shown at the bottom.

Sanger sequencing of RT-PCR products showed that three previously described patients, HBL97, HBL111, and HBL117 carry the same fusion transcript found in FLC patients (Figure 1A). Figure 1B–D show the analysis of biliary atresia patients. RT-PCR using specific primers detected the fusion transcript in three biliary atresia patients (Figure 1B), while three others were fusion-negative. Figure 1C shows fusion transcripts detected by RNA sequencing in patient BA3, but not in fusion-negative BA1. Figure 1D shows that RNA-Seq of transcripts from patients with BA 2, 4 and 5 is identical to transcript from the lung metastasis of FLC patient #58. These findings confirm our previous Western blot-based detection of the fusion kinase in these BA patients, as reported in our *Cancers* paper [9].

*RT-PCR-Sequencing and Immunostaining Analyses Revealed That DNAJB1::PRKACA Is Detected in Liver Tumors of Recently Collected Specimens from HBL and HCN-NOS Patients*

**Figure 2:**
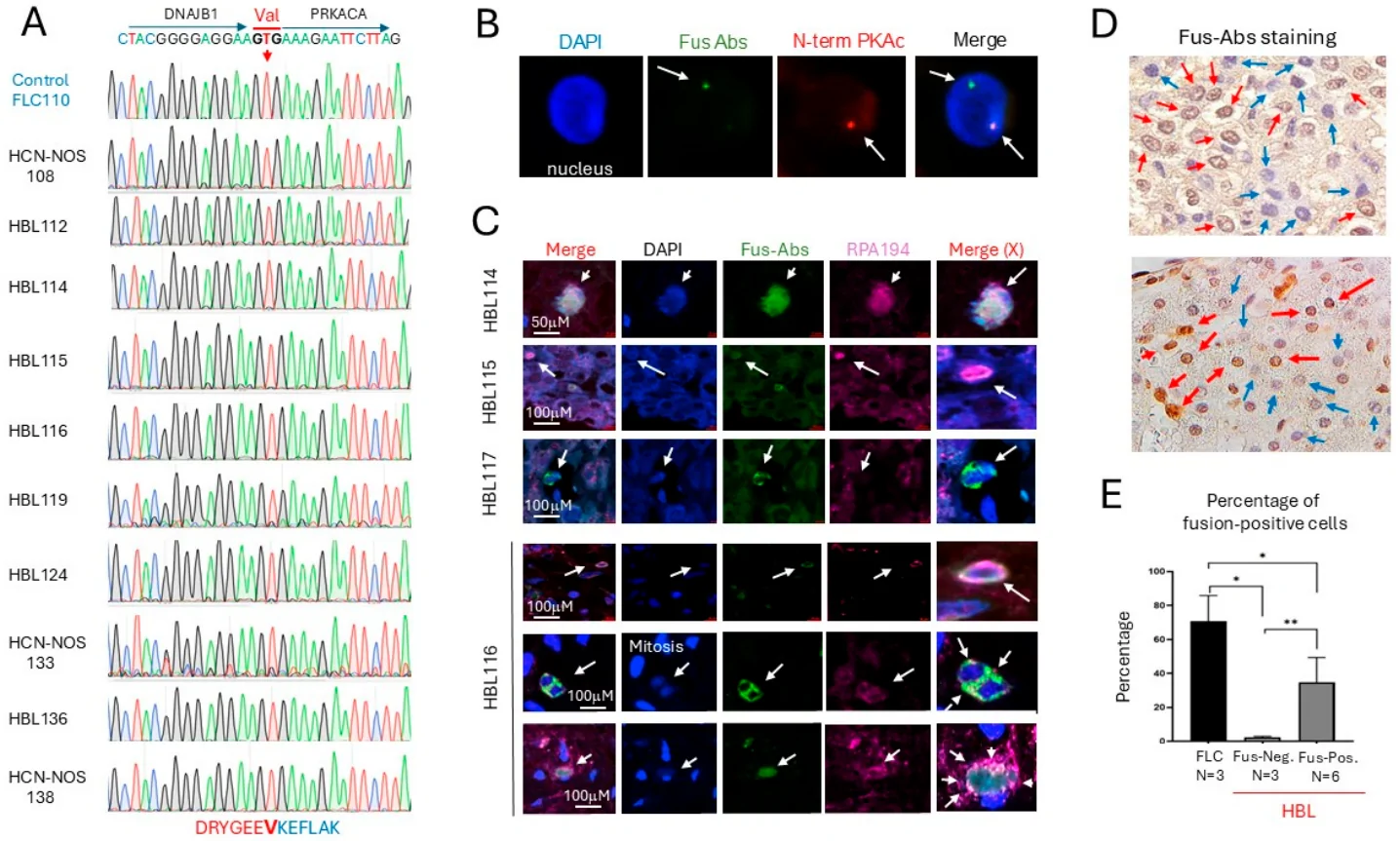
DNAJB1::PRKACA kinase is expressed in new pediatric liver tumors. (**A**) DNA sequencing results of RT-PCR fusion transcripts (nucleotide pick alignment) are shown for patient FLC110 (control) and HBL and HCN-NOS patients 108, 112, 114, 115, 116, 119, 124, 133, 136 and 138. The fusion part (GTG/Val) is shown above. The amino acid sequence of the fusion region, identified by translate-bioinformatics analysis and containing the peptide used to generate Fus-Abs, is shown at the bottom. (**B**) Confocal microscopy illustrates a nucleus within an FLC tumor, co-stained with Fus-Abs (green) and with antibodies targeting the N-terminus of PKAc (red), which do not detect the fusion kinase. (**C**) Confocal microscopy: Representative co-staining images show Fus-Ab signal with RNA Pol I (RPA194), used as a nuclear marker, in fusion-positive HBL114, HBL115, HBL117, and HBL116 samples. Merge X displays enlarged merged images highlighting the colocalization of nuclear signals. Scale bars are shown. (**D**) Example showing fusion-positive (red arrows) and fusion-negative (blue arrows) cells with nuclear DNAJB1::PRKACA in HBL#40. (**E**) Bar graphs summarize nuclear DNAJB1::PRKACA signals in fusion-positive HBL/HCN-NOS samples (N = 6), FLC patients (N = 3), and fusion-negative HBLs (N = 3). *, ** indicate *p* values of <0.05 and <0.01, respectively, calculated using Student’s *t*-test.

Our prior research has shown that some patients with aggressive pediatric liver cancers, such as HBL and HCN-NOS, have expressed the fusion kinase [9]. Additionally, we noted that those with fusion-positive HBL/HCN-NOS tend to have more aggressive diseases, characterized by increased fibrosis, resistance to chemotherapy and the development of lung metastases; some also exhibit fatty liver features [9]. After publishing our work in *Cancers*, we collected new samples and analyzed them for the expression of DNAJB1::PRKACA. Figure 2A presents nucleotide peaks of sequencing of RT-PCR products from FLC110 (the control), as well as new HBL/HCN-NOS patients # 108, 112, 114, 115, 116, 119, 124, 133, 136 and 138. As shown, the RT-PCR product sequences of these HBL/HCN-NOS match FLC110 and all include the fusion region targeted by Fus-Abs as found by the “translate-bioinformatic” program (see below).

We also calculated the number of fusion-positive cells in fusion-positive HBL samples and compared them with FLC patients. We focused on nuclear fusion signals because nuclei provide discrete, readily identifiable structures and a higher signal-to-noise ratio. In contrast, cytoplasmic signals may be heterogeneously distributed and more difficult to distinguish from background. We also found that the fusion kinase has a nuclear function (manuscript in preparation). Immunostaining was performed using Fus-Abs, whose specificity was described in our previous studies [9,11]. Before quantifying nuclear Fus-positive cells, we further characterized Fus-Ab specificity for nuclear staining. To assess whether Fus-Abs detect the fusion kinase rather than native PKAc, we used confocal microscopy with Fus-Abs and antibodies against the N-terminal peptide of native PKAc in a human FLC tumor sample (FLC110). Although native PKAc is primarily cytoplasmic, it is occasionally detected in the nucleus. As shown in Figure 2B, nuclear DNAJB1::PRKACA fusion kinase and native PKAc are both detectable, but they do not overlap. To confirm nuclear localization of DNAJB1::PRKACA in tumor sections from HBL/HCN-NOS patients, we co-stained the fusion kinase with the nuclear marker RNA Polymerase I (RPA194) (Figure 2C). Fusion-positive nuclear signals co-localized with RPA194 in tumor sections from four fusion-positive HBL patients. The proportion of DNAJB1::PRKACA nuclear-positive cells varied among samples, including those with high fusion-kinase levels. Figure 2C (bottom) shows DNAJB1::PRKACA and RPA194 staining in HBL116, which expresses high levels of the fusion kinase. Overall, our findings indicate that DNAJB1::PRKACA is present in the nuclei of HBL/HCN-NOS patients with aggressive liver cancer features.

We then quantified the percentage of cells with nuclear DNAJB1::PRKACA positivity. In HBL #40, approximately 35–42% of tumor cells were nuclear DNAJB1::PRKACA-positive (red arrows, Figure 2D). Fusion-negative cells are marked with blue arrows. We applied this analysis to three fusion-negative and six RT-PCR-confirmed fusion-positive HBLs. Overall, about 38% of cells from HBL/HCN-NOS patients showed elevated nuclear DNAJB1::PRKACA (Figure 2E). We also compared fusion-positive cell numbers in FLC patients and fusion-positive pediatric HBL/HCN-NOS tumors. FLC tumors contained approximately 70–80% fusion-positive cells, whereas HBL/HCN-NOS tumors with high DNAJB1::PRKACA levels averaged at about 38% fusion-positive cells (Figure 2E).

*RNA Transcripts of the Fusion-Positive HBL-HCN-NOS and BA Patients Code for Amino Acid Peptide That Is in Use for Trial Phase 1 for FLC Patients*

**Figure 3:**
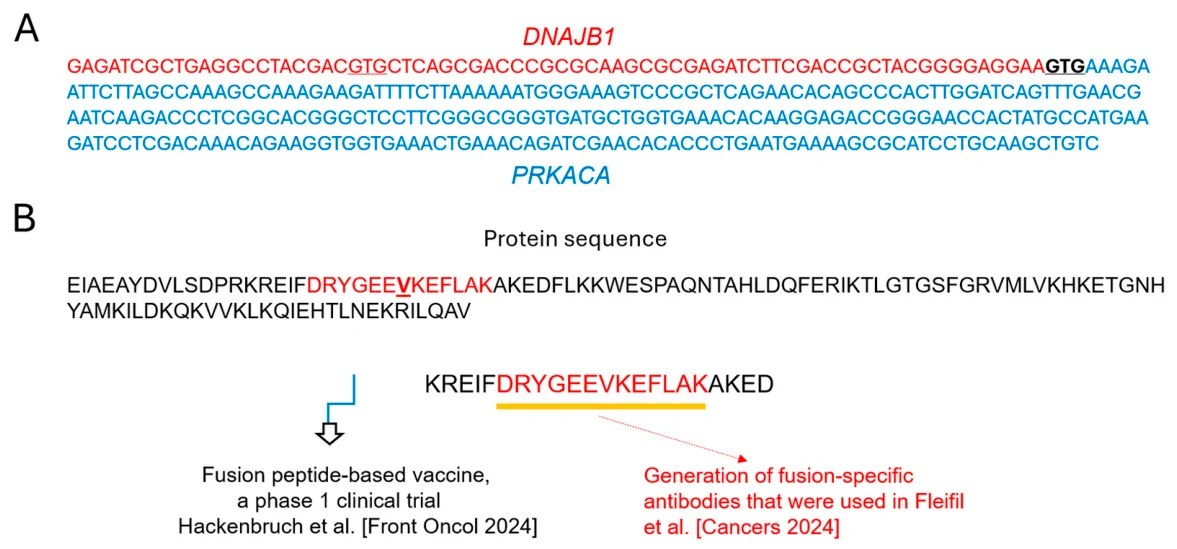
(**A**) NCBI BLAST analysis of RT-PCR products. GTG codon connects the DNAJB1 sequence and PRKACA sequence. (**B**) The RT-PCR product nucleotide sequences were translated into protein sequences using the “translate-bioinformatics” program. Bottom: The sequence of the peptide used in a fusion peptide-based vaccine clinical trial [12]. The peptide highlighted in red was used to create fusion-specific antibodies, as described in our publications [9,11].

Next, we looked at the amino acid sequences of the peptides encoded by RT-PCR products of fusion-positive HBL/HCN-NOS and biliary atresia patients. NCBI BLAST analysis of RT-PCR products revealed that DNAJB1 and PRKACA fuse at the GTG site, producing valine as the connecting amino acid for these sequences (Figure 3A). Entire DNAJB1::PRKACA transcripts produce a long fusion peptide in FLC, HBL, and BA patients, as shown by translation of DNA to protein sequences (Figure 3B). The protein sequences of RT-PCR products also include a short fusion peptide, which was utilized to produce fusion-specific antibodies [9,11]. As shown in Figure 3B (bottom) this same fusion peptide was also incorporated into a vaccine evaluated during a phase 1 clinical trial for FLC [12]. In summary, these data provide additional molecular support for the ongoing clinical trial and for the generation of the antibody that recognizes the fusion protein [9,11]. It should be noted that Figure 2 and Figure 3 present a portion of our studies of HBL/HCN-NOS patients using Sanger sequencing. A detailed examination of these patients is included in a new manuscript which is in preparation (Gulati & Timchenko).

*Limitations of Combining HBL and BA RNA-Seq Datasets Generated with Different Designs*

Palaz and colleagues analyzed RNA-Seq datasets of HBL, BA and FLC with Arriba, a fusion-calling tool for RNA-Seq data [13]. Using this tool and available RNA-Seq datasets, the authors did not find DNAJB1::PRKACA in HBL and BA patients. Based on our review of this paper, we propose a few potential explanations for why DNAJB1::PRKACA was not identified in RNA-Seq datasets from HBL and BA.

One likely reason is the variation in preparation of RNA-Seq datasets. An RNA-Seq protocol is designed based on investigators’ specific goals [14]. The key difference in RNA-Seq preparation is the “read length.” Preparation of RNA-Seq for gene expression analysis uses single-end 50 bp and paired 50–100 bp reads, while RNA-Seq datasets for splicing and fusion detection are usually prepared with paired 150 bp read length [14]. In the commentary, the analyzed RNA-Seq datasets of HBL and BA samples used 50 bp single-end read length and 50–100 bp paired read length (see Table 1 in Palaz’s commentary [13]). However, cited FLC reports used RNA-Seq datasets with 150 bp paired read length. We noted that Palaz’s commentary cited studies reporting other fusion events in RNA-Seq datasets with 50–100 bp reads (references 17–19 in Palaz’s commentary [13]) to support comparisons between FLC and HBL/BA datasets. However, these studies advise against using short-read RNA-Seq to compare fusion events. Specifically, reference 17 in Palaz’s commentary [13] (ref. [15] in our Reply) examined how read length affects fusion-prediction accuracy using five datasets with 50 bp reads and five with 101 bp reads. Across different methods, the authors reported that “all methods had improved accuracy with longer vs. shorter reads.” The authors also stated that “fusion detection sensitivity was affected by fusion expression level. Most methods were more sensitive at detecting moderately and highly expressed fusions but differed substantially in their ability to detect lowly expressed fusions” [15]. Although these limitations are especially important for such comparisons, they were not considered in either Palaz’s or Arif’s commentary [16].

Notably, a recent meta-analysis by Ostrowska and Gambin assessed RNA-Seq fusion detection tools and cautioned against directly comparing datasets generated by different sequencing methods [17]. The authors further noted that single-end sequencing is unsuitable for this purpose. For paired-end sequencing, they found that “shorter read lengths, such as 50 bp, were less effective for accurately identifying gene fusions. In some cases, certain tools did not support analysis of 50 bp samples at all.” Despite these established limitations, Palaz’s commentary compared HBL and BA datasets generated for gene expression analysis (single-end 50 bp and paired-end 50–100 bp reads) with FLC datasets optimized for fusion detection (paired-end 150 bp reads; Table 1 in Palaz’s commentary). In summary, comparing DNAJB1::PRKACA detection across RNA-Seq datasets prepared for gene expression analysis versus fusion detection is problematic and may lead to inaccurate results and conclusions.

Another issue in the design and conclusions of Palaz’s commentary concerns the use of Arriba as a tool for comparisons. Although Arriba is a recognized fusion-detection tool, it is not the most effective option. Tian and colleagues developed CICERO (Clipping Extended for RNA Optimization), a fusion-gene detection program that leverages longer next-generation sequencing (NGS) reads [18]. The authors reported that “CICERO detected 95% of the driver fusions with an average ranking of 1.9, whereas ChimeraScan, deFuse, FusionCatcher, STAR-Fusion, and Arriba detected only 63%, 66%, 77%, 63%, and 78%.” Thus, Arriba likely missed the DNAJB1::PRKACA fusion event in the HBL and BA RNA-Seq datasets because of its lower sensitivity.

The second reason the commentaries may not have detected DNAJB1::PRKACA in HBL and BA RNA-Seq data is the heterogeneity of these patient groups, which include both fusion-negative cases and cases with low DNAJB1::PRKACA expression. In contrast to the more uniform FLC RNA-Seq datasets, in which all patients showed high DNAJB1::PRKACA levels, HBL and BA datasets are more variable. In this regard, Haas’s report noted that “Fusion detection sensitivity was affected by fusion expression level” [14]. Therefore, Arriba may have lacked the sensitivity needed to detect low DNAJB1::PRKACA levels in the examined HBL and BA RNA-Seq datasets generated for gene-expression analysis.

Summary/Conclusions: Although our *Cancers* paper identified expression of the DNAJB1::PRKACA in HBL and BA samples [9], two commentaries could not detect the fusion in available RNA-Seq datasets of HBL and BA [13,16]. After analyzing these commentaries, we believe the main reason for this discrepancy is that they analyzed heterogenous HBL samples in RNA-Seq datasets that were originally generated for the examination of gene expression, not for the detection of fusion transcripts. We have also utilized multiple sensitive methods such as RT-PCR, Sanger sequencing, protein analysis via Western blot, and immunofluorescence analysis to analyze each sample individually, rather than as a group average. The reason to examine each case separately was to determine the variability in fusion expression in our HBL cohort, including the samples with no DNAJB1::PRKACA fusion kinase, samples with low levels of DNAJB1::PRKACA fusion kinase, and samples with high levels of the DNAJB1::PRKACA fusion kinase. In contrast to our sensitive approaches with individual HBL and BA patients, the two commentaries used combined RNA-sequencing data from studies that were originally not intended to compare fusion DNAJB1::PRKACA expression in FLC and HBL and failed to account for fusion expression heterogeneity in HBL cases. It is also important to evaluate both the DNAJB1::PRKACA fusion transcript and fusion protein in samples from individual HBL patients, because the DNAJB1::PRKACA protein (not RNA or DNA) is the main driver of pathological changes in FLC and fusion-positive HBL. This was not addressed by experimental work in the commentaries by Palaz and Arif. In this context, a recent report has examined a mouse model of IOPN with overexpression of DNAJB1::PRKACA protein and provided clear evidence that DNAJB1::PRKACA protein caused pathological changes in non-FLC IOPN-related liver disease and demonstrated a critical role for DNAJB1::PRKACA in IOPN-associated cholangiocarcinoma and pancreatic carcinoma [19]. Together, our data and this recent report support the conclusion that the DNAJB1::PRKACA fusion kinase occurs in non-FLC cancers and contributes to their development. Given the growing number of reports describing DNAJB1::PRKACA expression in non-FLC cancers, researchers should use caution when considering DNAJB1::PRKACA as a marker of FLC. Regarding further studies of pediatric liver cancer, the implementation of fusion immunodetection, fusion RT-PCR analysis, and RT-PCR-Sanger sequencing with each individual patient enables accurate identification of fusion transcripts and protein in HBL and BA cases as shown in Figure 2.

## Data Availability

All data are included in this manuscript.

## Abbreviations

FLC—fibrolamellar hepatocellular carcinoma; HBL—hepatoblastoma; DNAJB1::PRKACA, DNAJB1-PKAc or J-PKAc—the fusion kinase.
